# Characterization and Potential Function Analysis of the *SRS* Gene Family in *Brassica napus*

**DOI:** 10.3390/genes14071421

**Published:** 2023-07-10

**Authors:** Ming Hu, Meili Xie, Xiaobo Cui, Junyan Huang, Xiaohui Cheng, Lijiang Liu, Shunping Yan, Shengyi Liu, Chaobo Tong

**Affiliations:** 1 College of Life Science and Technology, Huazhong Agricultural University, Wuhan 430070, China; huming199217@126.com (M.H.);; 2 Key Laboratory of Biology and Genetics Improvement of Oil Crops, Oil Crops Research Institute of Chinese Academy of Agricultural Sciences, Ministry of Agriculture and Rural Affairs, Wuhan 430062, China; xiemeili0101@163.com (M.X.); cuixb223@outlook.com (X.C.); huangjy@oilcrops.cn (J.H.); liusy@oilcrops.cn (S.L.)

**Keywords:** *SRS* gene family, *Brassica napus*, expression pattern, agronomic traits

## Abstract

SRS (SHI-related sequence) transcription factors play a crucial role in plant growth, development, and abiotic stress response. Although *Brassica napus* (*B. napus)* is one of the most important oil crops in the world, the role of *SRS* genes in *B. napus* (*BnSRS*) has not been well investigated. Therefore, we employed a bioinformatics approach to identify *BnSRS* genes from genomic data and investigated their characteristics, functions, and expression patterns, to gain a better understanding of how this gene family is involved in plant development and growth. The results revealed that there were 34 *BnSRS* gene family members in the genomic sequence of *B. napus*, unevenly distributed throughout the sequence. Based on the phylogenetic analysis, these *BnSRS* genes could be divided into four subgroups, with each group sharing comparable conserved motifs and gene structure. Analysis of the upstream promoter region showed that *BnSRS* genes may regulate hormone responses, biotic and abiotic stress response, growth, and development in *B. napus*. The protein-protein interaction analysis revealed the involvement of *BnSRS* genes in various biological processes and metabolic pathways. Our analysis of *BnSRS* gene expression showed that 23 *BnSRS* genes in the callus tissue exhibited a dominant expression pattern, suggesting their critical involvement in cell dedifferentiation, cell division, and tissue development. In addition, association analysis between genotype and agronomic traits revealed that *BnSRS* genes may be linked to some important agronomic traits in *B. napus*, suggesting that *BnSRS* genes were widely involved in the regulation of important agronomic traits (including C16.0, C18.0, C18.1, C18.2 C18.3, C20.1, C22.1, GLU, protein, TSW, and FFT). In this study, we predicted the evolutionary relationships and potential functions of *BnSRS* gene family members, providing a basis for the development of *BnSRS* gene functions which could facilitate targeted functional studies and genetic improvement for elite breeding in *B. napus*.

## 1. Introduction

*B. napus* (AACC, 2n = 38), also known as rapeseed, is a significant industrial crop and a popular source of edible oil source globally and originated about 7500 years ago from a cross between two diploid species, *B. rapa* (AA, 2n = 20) and *B. oleracea* (CC, 2n = 18) [1]. The ancestor of *B. napus* underwent triploidization before hybridization, resulting in multiple duplication events throughout its evolutionary history. As a result, the copy number of the gene from *B. napus* should be six times that of *Arabidopsis*. *B. napus* serves multiple purposes in our daily lives, including being used as edible oil, vegetable, high-quality protein animal feed, potential energy crop, and so on. *SRS* transcription factors play an important role in various biologic processes [2,3,4,5,6,7,8,9,10]. Nevertheless, their function in *B. napus* has not been extensively studied.

The *SRS* transcription factors constitute an ancient gene family in plants that are pivotal in diverse biological processes including plant growth, development, hormone synthesis, and signal transduction [11,12]. The majority of members in this family share two constant domains, namely the N-terminal RING-like zinc finger and the C-terminal IGGH domain [12,13]. The RING-like zinc finger domain contains a conserved cysteine-rich ring finger domain (RING finger domain), which engages in various physiological and biochemical processes by acting as E3 ubiquitin ligase or transcription factors [14]. Another IGGH domain, which is abundant in acidic amino acids, promotes the creation of both homo- and heterodimer protein complexes [13]. In *Arabidopsis*, the gene family’s original discovery site, it has been observed to exert a negative influence on root development by modulating the biosynthesis of the auxin hormone [3].

The number of *SRS* gene family members differs among different plants. *Arabidopsis thaliana* (*Arabidopsis*), for example, a popular model plant, contains 10 members including *SHI*, *STY1*(*SRS1*/*STYlISH1*), *STY2*(*SRS2*/*STYlISH2*), *LRP1*(LATERAL ROOT PRIMORDIUM1), and *SRS3*–*SRS8* (SHI-related sequence 3–8) [2,4,5,6], 6 in rice [7], 26 in cotton [8], and 21 in soybean [9]. Extensive research has been conducted on *SRS* genes since their discovery in *Arabidopsis*. Studies have shown that *SRS* genes can regulate root development by inducing the expression of the auxin hormone synthesis gene *YUCCA4*. Moreover, when the *LPR1* gene is overexpressed, auxin hormone levels rise, inhibiting root development [2,4]. Both leaf and flower tissues play crucial roles in the vegetative and reproduction process. A large number of loci associated with the development of these tissues have been identified in *Brassica*, such as the *FLC* gene [14], the *MADS-box* genes [15], the *BoPLD1* gene [16], the *BnDWF/DCL1* locus [17], *BoABI1*, *BoAP1*, *BoPLD1*, *BoTHL1* and *PBCGSSRBo39* [18], and the *Ll 3.2* locus [19]. In addition, *SRS* genes can also affect leaf and flower development. Mutations in a single *AtSRS* gene can lead to altered leaf shape and abnormal flower development, and when multiple *SRS* genes are mutated simultaneously trait differences also increase, indicating some functional overlap among the *SRS* family members [10,12]. Overexpressing the *AtSHI* gene results in significant dwarfism and delayed flowering [11,20]. Meanwhile, in barley, *SRS* genes can regulate awn length and flower development [21,22]. While in maize, *SRS* genes could take part in carbohydrate redistribution during leaf senescence [23]. Abiotic stresses such as drought, temperature, salt, and nutrient stresses can alter plant biosynthesis and nutrient acquisition throughout plant growth and development. These stresses emerge as significant factors that restrict plant growth and impact crop yield and quality [24,25,26]. In winter rapeseed, a large number of stable reference genes have been identified under various stresses by integrating multiple tools under different stress [27]. The co-expression of multiple genes can improve stress tolerance for better adaptation to different abiotic stresses in *B. napus* [28]. Glucobrassicin (GBS), a secondary metabolite found in a large number of *Brassica* species, exhibits an increase in content under various abiotic stresses [29]. Furthermore, *SRS* genes also showed important roles during salt-stressed environments, indicating that they are also involved in the biological processes of adversity stress [9,30].

The *SRS* gene family plays a vital role in various biological processes such as plant growth, hormone regulation, stress response, and development. A comprehensive understanding of the *SRS* gene family`s functions can assist in breeding superior plant varieties. Nevertheless, there is limited information available on the *SRS* gene family in *B. napus*. Therefore, this study employs a bioinformatic approach to characterize and investigate the *BnSRS* gene family’s structure, expression, distribution, evolutionary patterns, and potential impact on agronomic traits, aiming to provide a better understanding of its potential function and assist in the breeding of superior varieties of *B. napus* in the future.

## 2. Materials and Methods

**Figure 1:**
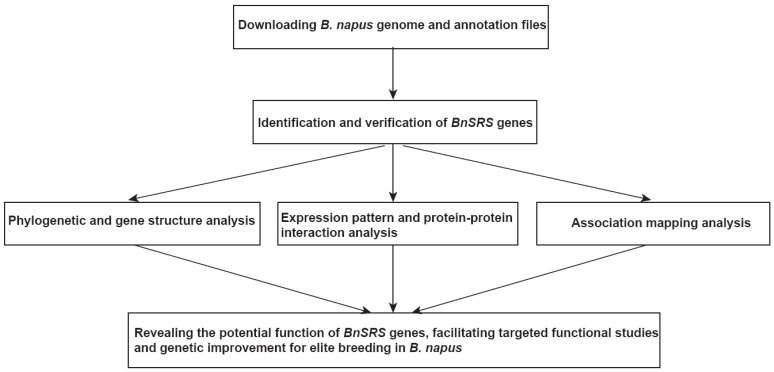
The pipeline for *BnSRS* gene family analysis.

To provide a comprehensive overview of the study, a flow chart was created (Figure 1). In detail, we performed the identification and verification of the *BnSRS* gene after downloading the genome sequence and annotation files. Once the *BnSRS* genes were obtained, evolutionary and gene structural analysis, expression pattern analysis, protein-protein interacting analysis, and association mapping analysis were performed to reveal the potential function of the *BnSRS* genes, facilitating targeted functional studies and genetic improvement for elite breeding in *B. napus*.

### 2.1. Characterization and Physicochemical Characterization of BnSRS Family Members

The analysis utilized the commonly used reference genome, Darmor-*bzh* (v4.1) [1], which is the first assembled *B. napus* genome based on high-depth sequencing data, with a genome size of approximately 850 Mb, containing 101,040 protein-coding genes. The genome sequence and gene structure files were obtained from the BRAD database (http://brassicadb.cn/, accessed on 6 March 2023). The SRS protein sequence of the model plant *Arabidopsis* was downloaded from the TAIR database (https://www.arabidopsis.org, accessed on 6 March 2023). To identify the *BnSRS* genes, the Hidden Markov Models (HMM) file, PF05142, of the *SRS* gene family was downloaded from the Pfam database (v35.0, http://pfam.xfam.org, accessed on 6 March 2023) [31]. *BnSRS* candidate genes were identified by searching through the *B. napus* protein sequence using hmmer3.0 software with a set e-value < 1 × 10^−5^ [32]. The candidate gene was further verified against the conserved domain using the online web tool NCBI-CDD (https://www.ncbi.nlm.nih.gov/Structure/cdd/docs/cdd_search.html, accessed on 6 March 2023) [33] and the SMART database (http://smart.embl.de/, accessed on 6 March 2023) [34] with a default parameter. The remaining *BnSRS* genes were subjected to the online software ExPASy (http://web.expasy.org/protparam/, accessed on 6 March 2023) to predict the molecular weights (MW), isoelectric point (pI), Instability Index, aliphatic index, and grand average of hydropathicity. Then, the subcellular location of BnSRS proteins was predicted using the WoLFPSORT online tool (https://wolfpsort.hgc.jp/, accessed on 6 March 2023) [35]. Finally, the position of the *BnSRS* gene on the chromosome was determined based on the reference genome annotation information.

### 2.2. The Phylogenetic Analysis of BnSRS

The SRS protein from *B. napus*, *Arabidopsis*, rice (*Oryza sativa*), cotton (*Gossypium hirsutum*, *G. hirsutum*), and soybean (*Glycine max*) were used to construct the phylogenetic tree. First, multiple sequence alignment was performed on the integrated protein sequences using MUSCLE software [36]. Next, MEGA software was used to construct a phylogenetic tree based on the ML method with a bootstrap value set to 5000 [37]. Finally, the phylogenetic tree file was uploaded to the online software iTOL v6.5.2 (https://itol.embl.de/) for visualization [38]. Multiple synteny analysis was also performed between these five species by using Tbtools software [39]. To identify the duplicated gene pairs of *BnSRS*, the software BLASTP was used to align the BnSRS protein sequences with the e-value of 1 × 10^−10^ [40], then the software MCScan X [41] was used to identify the duplicated gene pairs of *BnSRS* which were visualized using the software Circos [42]. Once the duplicated gene pairs of *BnSRS* were obtained, the software Tbtools was used to calculate the evolutionary pressure of each duplicated gene pair of *BnSRS* [39].

### 2.3. The Gene Structure, Conserved Motifs, and Cis-Acting Regulatory Elements Analysis

The exon and intron structures of the *BnSRS* gene were obtained based on the genome annotation file. The conserved motifs of the *BnSRS* gene family were predicted using the online software MEME (v5.5.3, https://meme-suite.org/tools/, accessed on 6 March 2023) with a maximum number of conserved motifs set to 10 [43]. To identify *cis*-acting regulatory elements, the sequences in the 2 kb upstream of the *BnSRS* genes were extracted, and then the online software PlantCARE5 (https://bioinformatics.psb.ugent.be/webtools/plantcare/html/, accessed on 6 March 2023) was used to predict the *cis*-acting regulatory elements of each *BnSRS* gene [44]. Finally, gene structure, conserved motifs, and *cis*-acting regulatory elements were visualized by Tbtools [39]. 

### 2.4. Analysis of the Expression Pattern of BnSRS in Different Tissues and Different Environments

Transcriptome data were obtained from the previous studies that included a total of 32 tissues containing bud, callus, leaf, stamen, new pistil, blossomy pistil, wilting pistil, stem, sepal, ovule, 11 time-course seeds and silique walls (0, 4, 8, 12, 16, 20, 24, 28, 32, 40, 48), and five stress conditions (*Sclerotinia sclerotiorum* (*S. sclerotiorum*), dehydration, salt, cold, and abscisic acid) [45,46,47]. These data were aligned to the reference genome by software hisat2 with the following parameters: -t -p 40—min-intronlen 20—max-intronlen 20,000—dta [48], and the expression quantity based on the transcripts per kilobase million (TPM) normalization method was calculated by software Stringtie with the setting: -e -B -p 30 -f 0.1 [49]. Finally, the expression of *BnSRS* was extracted and displayed by Tbtools [39].

### 2.5. Prediction of Protein-Protein Interaction Network Analysis

To explore proteins that interact with BnSRS proteins, based on the homologs of the *BnSRS* genes in *Arabidopsis*, we conducted a protein-protein interaction network analysis. These homologous proteins were subjected to the online software STRING (v12.0, https://www.string-db.org/, accessed on 6 March 2023) to obtain the interacting proteins [50]. Subsequently, the sequences of these proteins were aligned to obtain the homologous protein sequences in *B.napus*. Finally, these genes will be used to perform the GO and KEGG enrichment analysis by R package clusterProfiler to investigate the biological functions [51].

### 2.6. Association Mapping Analysis of BnSRS Genes with Important Agronomic Traits

To investigate the effect of the *BnSRS* gene on important agronomic traits, we used previously reported genotype data including 324 *B. napus* accessions [52]. SNPs within the *BnSRS* gene body were extracted and annotated using the software SnpEff [53]. Thereafter, 11 important agronomic traits were selected. These traits include fertility traits (final flowering time (FFT)). Proper fertility can improve the adaptability of *B. napus* to the season, which can be used to determine the area of *B. napus* promotion, and is very meaningful for achieving stable and high yields of *B. napus*. We also selected the yield trait of thousand seed weight (TSW), which is directly related to the yield of *B. napus*, and is of great importance for *B. napus* breeding. With the improvement of people’s living standards, people pay more and more attention to the quality of edible oil, therefore, we also investigated some quality traits (including palmitic acid (C16.0), stearic acid (C18.0), oleic acid (C18.1), linoleic acid (C18.2), linolenic acid (C18.3), eicosenoic acid (C20.1), erucic acid (C22.1), Glucosinolates (GLU), and protein). With the general linear model (GLM) method, an association mapping analysis was performed by the software rMVP between the SNP and these traits [54].

## 3. Results and Discussion

### 3.1. Identification of BnSRS Genes and Analysis of Physicochemical Properties of Its Family Members

The *SRS* gene family was represented by the Hidden Markov Model (HMM) PF05142, which was used to search for candidate genes with distinctive *SRS* domains in *B. napus* protein sequences. These candidate genes were verified through the online web tool NCBI-CDD [33] and SMART [34] database to identify genes with the DUF702 domain. As a result, 34 *BnSRSs*, named *BnSRS1*-*BnSRS34* according to their positions in the genome sequence, were identified. The number of *BnSRS* genes was six more than in the previous study, providing an excellent addition to the research of the *BnSRS* gene family [55]. The majority of the proteins encoded by these *BnSRS* genes (30/34) were found to be located in the nucleus (Table S1), which is in agreement with previous studies [55,56]. Additionally, various properties of these proteins such as the number of amino acids (AA), molecular weight (MW), isoelectric point (pI), instability index, aliphatic index, and grand average of hydropathicity were statistically analyzed using Table S1. The results showed that the length of the AA ranged from 130 to 346 with an average length of 280.9; the BnSRS29 protein was identified as the shortest, while the BnSRS32 protein was the longest. The MW of the proteins ranged from 15,066.46 to 38,329.85. The pI values were found to range from 4.56 to 10.06, with 13 pI values less than 7 and 21 pI values greater than 7, indicating that most of the BnSRS proteins were alkaline. The instability index ranged from 42.17–68.46, and all were unstable proteins. The aliphatic index ranged from 38.95–84.58. The grand average of hydropathicity ranged from −0.93 to −0.047, indicating that all the identified proteins were hydrophilic.

### 3.2. Phylogenetic Analysis of BnSRS

**Figure 2:**
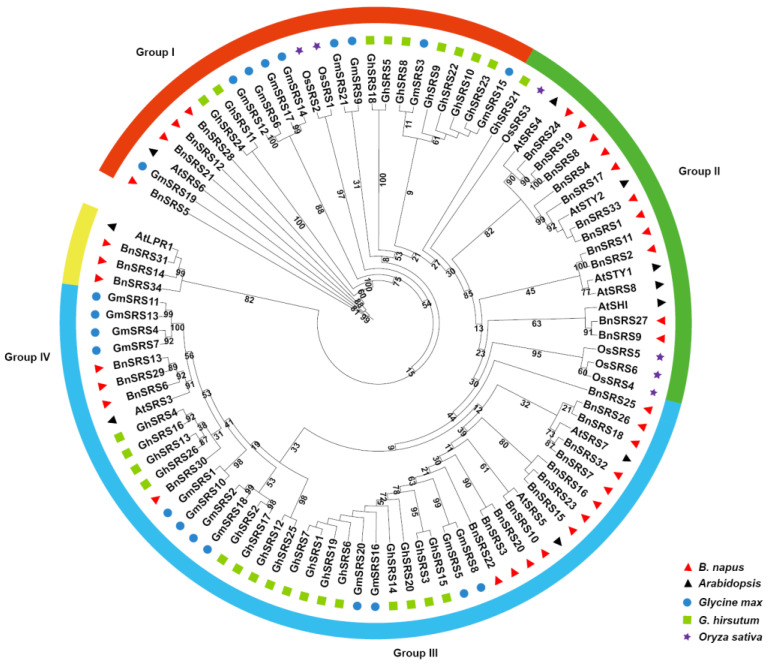
A phylogenetic tree was constructed using the ML method to analyze the relationship between *SRS* genes. The *BnSRS* genes were found to be clustered into four distinct subgroups, distinguishable by different colors in the tree.

To reveal the phylogenetic relationship of *BnSRS*, a maximum likelihood (ML) phylogenetic tree was constructed using protein sequences of the *SRS* gene from 34 *BnSRSs*, 10 *AtSRSs*, 6 *OsSRSs* [7], 26 *GhSRSs* [8], and 21 *GmSRSs* [9]. The results showed that all *SRSs* could be classified into four groups according to the clustering relationship, with varying numbers of gene members per group. Group I comprised 25 members, including 1 from *Arabidopsis*, 4 from *B. napus*, 2 from rice, 9 from cotton, and 9 from soybean. Group II comprised 21 members, including 5 from *Arabidopsis*, 11 from *B. napus*, 4 from rice, 1 from cotton, and none from soybean. The largest number of members was found in group III, which had 47 members comprising 3 from *Arabidopsis*, 16 from *B. napus*, none from rice, 16 from cotton, and 12 from soybean. Lastly, group IV comprised 4 members with 1 and 3 members in *Arabidopsis* and *B. napus*, respectively (Figure 2). Additionally, multiple synteny analysis was also performed among the five species (Figure S1), which revealed that a large and significant number of *SRS* genes were not homologous to *SRS* genes in other species, implying that *SRS* genes have undergone substantial divergence during the evolutionary process.

### 3.3. Analysis of Chromosomal Localization and Duplication Events of BnSRS Gene Family Members

**Figure 3:**
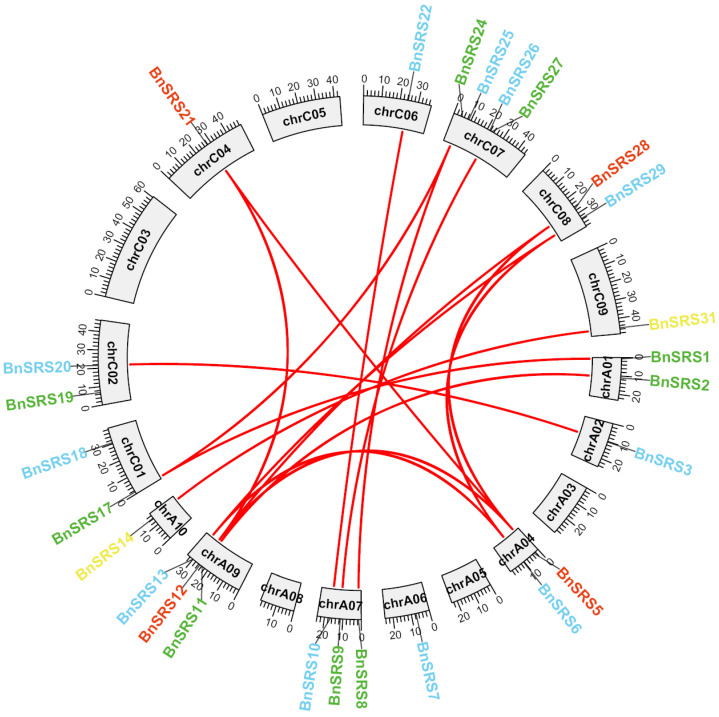
The distribution and collinearity of the *BnSRS* genes. Different color represents different subgroups, the red lines indicated the duplicated gene pairs of *BnSRS*.

Gene duplication is a widespread phenomenon among various species and plays a critical role in promoting species diversity and creating novel species [57,58]. This study focuses on *B. napus*, which is believed to have originated from natural hybridization between *Brassica rapa* and *Brassica oleracea* about 7500 years ago [1,59,60]. The ancestor of *B. napus* underwent triploidization before hybridization, resulting in multiple duplication events throughout its evolutionary history. As a result, the number of *BnSRS* gene families should be six times that of *Arabidopsis*. However, the annotation file revealed 34 members of the *BnSRS* gene family, which was lower than expected. This may be due to the loss of the *BnSRS* gene or functional divergence. Twenty-six *BnSRS* genes were unevenly distributed over 14 chromosomes, with the highest number of members found on chromosome C07, which contains 4 *BnSRS* members. Chromosomes A07 and A09 both had 3 each, while chromosomes A01, A04, C01, C02, and C08 had 2 members each, and chromosomes A02, A05, A10, C04, C06, and C09 had only 1 member each (Figure 3 and Table S1). *B. napus*, an allotetraploid crop, has undergone multiple duplication events. To further explore the evolutionary relationships of *BnSRS*, we identified the duplication events and the duplicated gene pairs. A total of 20 duplicated gene pairs were found in the *BnSRS* genes, with 2 pairs of both genes found within the A subgenome, 1 pair in the C subgenome, and the remaining 17 duplicated gene pairs between the 2 subgenomes (Figure 3 and Table S2). Upon analyzing these gene pairs, we found that all had non-synonymous to synonymous substitutions (Ka/Ks) values less than 1, similar to that in *Cassava* [61], indicating that they underwent purifying selection during the evolutionary process. In addition, 23 out of the 34 *BnSRS* genes had undergone WGD (whole genome duplication) or segmental, which indicated that the expansion of the *BnSRS* gene members mainly resulted from WGD or segmental events.

### 3.4. Analysis of BnSRS Gene Structure and Conserved Motifs

**Figure 4:**
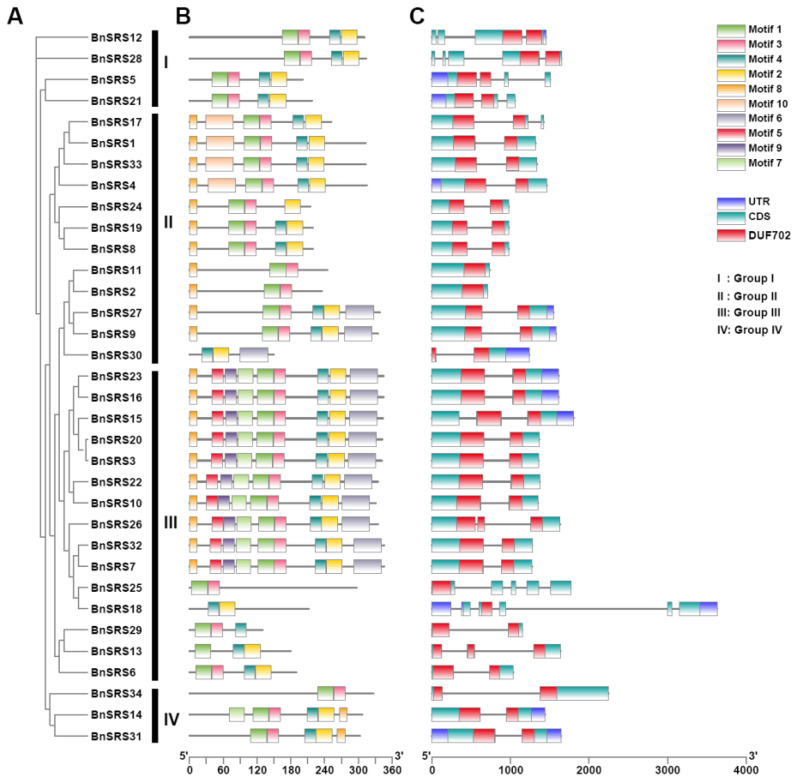
The phylogenetic tree, conserved motifs, and gene structure for the *BnSRS* genes. (**A**) The phylogenetic tree for the *BnSRS* genes was constructed. (**B**) The conserved motifs identified in the *BnSRS* genes. Different color represents different motif. (**C**) The gene structure of the *BnSRS* genes. The green, purple, and red box represent the CDS, UTR, and DUF702 domains, respectively. The red box is above the green box, and it covers part of the green box.

**Figure 5:**
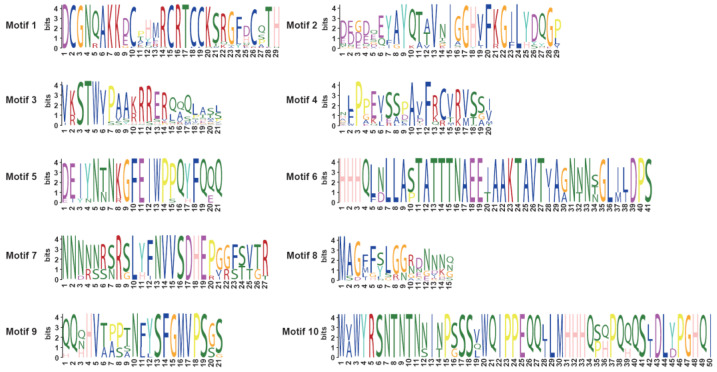
The sequence logos of conversed motifs in the BnSRS proteins. Each letter’s height depicts the occurrence frequency of the corresponding base or amino acid residue at that position.

The number of exons varied among subgroups. The highest average number of exons per gene was found in Group I (4). Followed by group II (average of 2, range of 1–3). Exon counts varied the greatest in group III, from 2 to 5, with an average of 3. Finally, group IV genes all contained 2 exons (Figure 4A,C). To analyze the domain functions present in the *BnSRS* genes, we analyzed their motif distribution. The results showed that the majority (32/34) of the *BnSRS* genes contained a RING-like zinc finger (motif 1), while 29 *BnSRS* genes had the IGGH domain (Motif 2), indicating that these two motifs tend to be conserved in the *BnSRS* gene family (Figure 5). In addition, different motifs were observed to have varying distributions among the different *BnSRS* subgroups. For example, motif 6 was only present in both group II and group III, while motif 10 was only present in group II, and motif 9 was only present in group III. The number of motifs also varied among subgroups, with group III having the highest number of motifs and group I having the fewest, suggesting that changes in motif number occurred during the evolutionary process of the *BnSRS* family members, leading to functional divergences among them (Figure 4B). In addition, similar motifs were observed among members of the same subgroups, suggesting potential similarities in evolutionary relationships or functions (Figure 2 and Figure 4).

### 3.5. Analysis of Cis-Acting Elements of the BnSRS Genes

**Figure 6:**
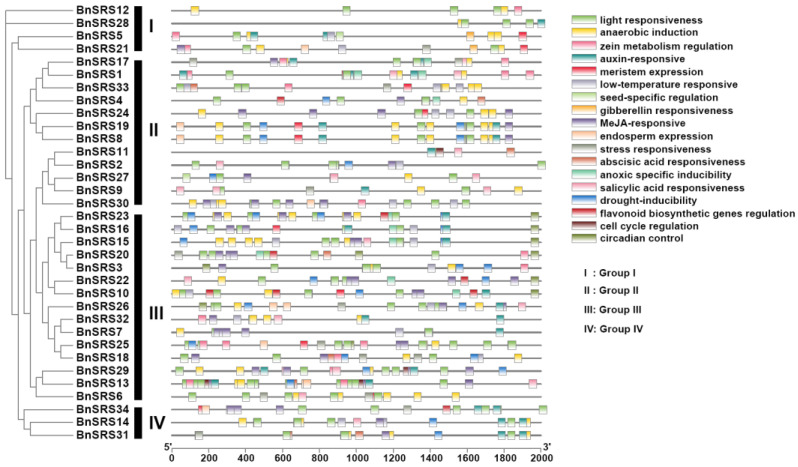
*Cis*-element analysis of the *BnSRS* genes. The different color boxes represent different *cis*-elements.

Plants have complex regulatory mechanisms that respond to biotic and abiotic stresses, and many of these mechanisms depend on *cis*-acting elements present in the promoter regions of genes [62,63]. The critical promoter region of a gene plays a crucial role in regulating its function [64]. To identify *cis*-acting elements of the *BnSRS* genes, a sequence of 2000 bp upstream of the translation starting at the site was extracted and submitted to PlantCare, an online web tool for *cis*-acting element analysis [44]. After analyzing the results, it was discovered that the extracted sequence contained various hormone-related elements, such as auxin-responsive, abscisic acid responsiveness, gibberellin responsiveness, MeJA-responsive, salicylic acid responsiveness, and others (AuxRR-core, ABRE, TATC-box, TGACG-motif, TCA-element). Additionally, stress-related elements, light responsiveness, low-temperature responsiveness, drought-inducibility, anaerobic induction, and others (TC-rich repeats, ACE, LTR, MBS, ARE) were also present. Furthermore, development-related elements were also identified, such as circadian control, cell cycle regulation, meristem expression, endosperm expression, and others (circadian, MSA-like, CAT-box, AACA_motif). Among these elements, ABRE, ARE, and CAT-box accounted for the highest number of hormone-related elements, stress-related elements, and development-related elements, respectively (Figure 6 and Table S3). All of the *BnSRS* genes’ promoter regions contained light-responsive elements, suggesting that the *BnSRS* genes may be involved in the light response during the growth and development of *B. napus*. The promoter regions of 30 *BnSRS* genes contained abscisic acid-responsive and anaerobic induction-related elements, while 26 *BnSRS* genes had MeJA-responsive related elements. Additionally, nearly half of the *BnSRS* genes (16/34) contained low-temperature responsive elements (Figure S2 and Table S3). Additionally, some *BnSRS* genes contained stress-responsive, drought-inducibility, cell cycle regulation, and other related elements in the promoter region (Figure 6). These results suggest that *BnSRS* genes may play a crucial role in regulating hormone responses and in response to stresses, as well as growth and development, which is consistent with the *SRS* gene family in *Cassava* [61] and cotton [8].

### 3.6. Expression Pattern Analysis of BnSRS Gene Family Members

**Figure 7:**
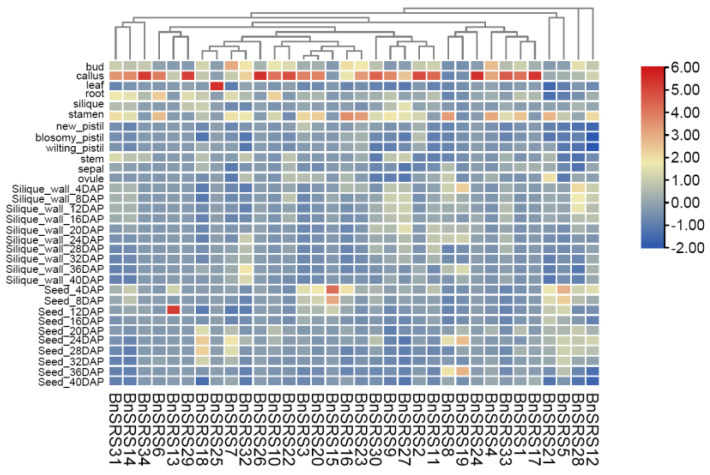
The expression levels of the *BnSRS* genes in 32 tissues. The red and blue represent high and low expressions, respectively. DAP represents days after pollination.

**Figure 8:**
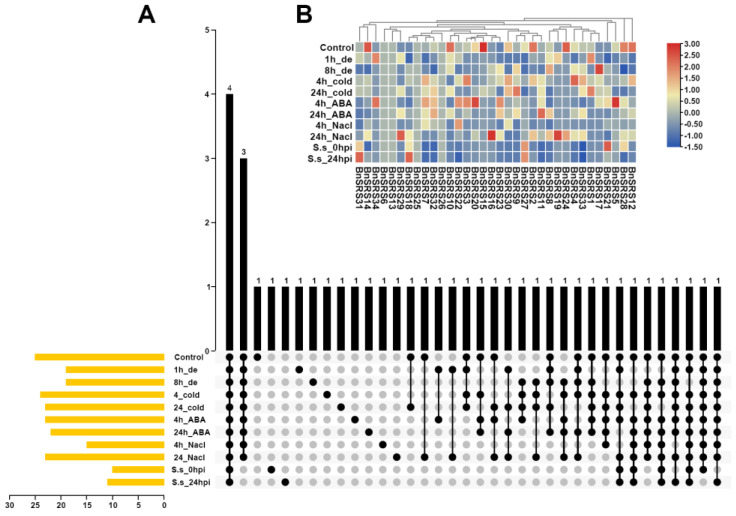
The expression pattern of the *BnSRS* genes under different biotic and abiotic stresses. (**A**) The number of *BnSRS* genes simultaneously expressed under different stresses. The ‘de’ represents dehydration and “S.s” represents *S. sclerotiorum*. The yellow bar represented the number of genes with expression in the tissue, the black dot indicated expression in the corresponding tissues, and the black bar indicated the number of genes expressed in the corresponding tissues. (**B**) The expression levels of the *BnSRS* genes under different stresses. The red and blue represent high and low expressions, respectively.

The expression pattern of genes could be used to infer the molecular functions of genes. Previous studies have shown that *SRS* genes were expressed in roots, stems, leaves, flowers, seeds, and other tissues, and were involved in growth and developmental processes in plants [2,3,6,7,8,9,11,20,23,56,65]. To further explore the function of the *BnSRS* gene family, we analyzed their expression patterns in different tissues and under various stresses. The results showed that only 2 *BnSRS* genes (*BnSRS18* and *BnSRS21*) were expressed in all 32 tissues, while 34 *BnSRS* genes were expressed in 1 or more tissues. Twenty-three of them showed a dominant expression in callus tissue; indicating the potential importance of *BnSRS* genes in cell division, growth, and development (Figure 7). In the silique wall tissue, only 14 *BnSRS* genes were expressed at all times. In addition, the expression of *BnSRS18* increased as DAP increased, except at 40 DAP, which showed a decrease (Figure S3A). Meanwhile, the expression of *BnSRS21* declined continuously with the increase of DAP (Figure S3A). In the seed tissue, the expression of *BnSRS21* showed a similar pattern to that in the silique wall tissue; while the expression of *BnSRS18* increased and then decreased, with the highest expression at the 28th DAP (Figure S3B). Moreover, *B.napus* is exposed to a variety of biotic and abiotic stresses during its growth and development, and *SRS* genes play an important role in response to biotic and abiotic stresses [7]. So, we also investigated the changes in *BnSRS* gene expression under biotic and abiotic stresses. The results showed that only 4 *BnSRS* genes had expression under all stresses (Figure 8A). The overall findings demonstrated that only 9 *BnSRS* genes were expressed under all abiotic stresses, and most of the genes were expressed at a low level under different abiotic stresses, except for *BnSRS18*, which demonstrated up-regulation under different abiotic stresses except under dehydration for 1 h, with the highest expression up-regulation observed under salt treatment for 24 h, which was more than 2-fold. Most *BnSRS* genes displayed significant down-regulation under diverse abiotic stresses, with *BnSRS2* showing the greatest decrease at 22-fold under ABA treatment for 24 h (Figure 8B). To investigate the expression profile of the *BnSRS* genes under biotic stress, we investigated the inoculation of *B. napus* at 0 and 24 h with *S. sclerotiorum*. The results showed that only some *BnSRS* genes responded to *S. sclerotiorum* inoculation (Figure 8B). Only the expression of *BnSRS18* showed a significant increase, with a nearly 2-fold up-regulation, while the expression of *BnSRS21* showed a substantial decrease, with a nearly 3-fold down-regulation (Figure 8B). These results suggested that the *BnSRS* genes play an important role in response to a variety of stresses, as in other species [6,7,8,9,61].

### 3.7. Prediction of BnSRS Proteins Interactions Analysis

**Figure 9:**
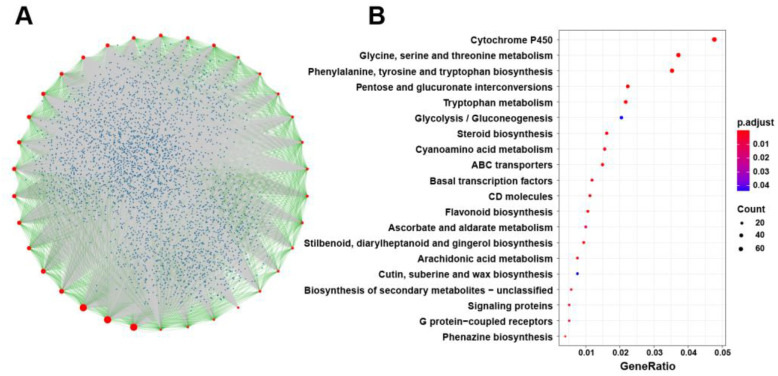
Protein-protein interaction analysis of *BnSRS* gene family members. (**A**) The network of protein-protein interaction analysis. The red points represent the *BnSRS* proteins, the blue points represent the proteins that interacted with the *BnSRS* proteins, the green lines represent the interactions between the *BnSRS* proteins, and the grey lines represent the interactions between the BnSRS proteins and the other proteins. (**B**) The KEGG pathway analysis of the genes encoding the proteins interacted with *BnSRS* genes.

To investigate the function of *BnSRS* genes in *B. napus*, we conducted a protein-protein interaction analysis using their homology to Arabidopsis. The results revealed that 20,145 genes interacted with these 34 *BnSRS* genes (Table S4). In the interaction network, these 34 BnSRS proteins were located in the central node, with most of them interacting with other proteins of *BnSRS* (Figure 9A). Furthermore, we performed the Gene Ontology (GO) enrichment and Kyoto Encyclopedia of Genes and Genomes (KEGG) pathway analysis on these interacting proteins. The KEGG pathway analysis showed that the genes encoding the interacting proteins were involved in various biochemical pathways, including phenylalanine, tyrosine and tryptophan biosynthesis, cytochrome P450, glycine, serine, and threonine metabolism, and so on (Figure 9B and Table S5). The GO enrichment analysis revealed that the genes encoding the interacting proteins were involved in multiple biological processes, such as transition metal ion transport, auxin-activated signaling pathway, stamen development, and so on. Furthermore, in terms of cellular components, these proteins were enriched in myosin complex, endosome membrane, microtubule, and others. In terms of molecular function, these proteins were enriched in tryptophan synthase activity, motor activity, pectinesterase activity, and so on (Table S6). Protein-protein interactions analysis showed that BnSRS proteins played an important role in many aspects of development and growth.

### 3.8. Genetic Effects of BnSRS Genes on B. napus Agronomic Traits

**Figure 10:**
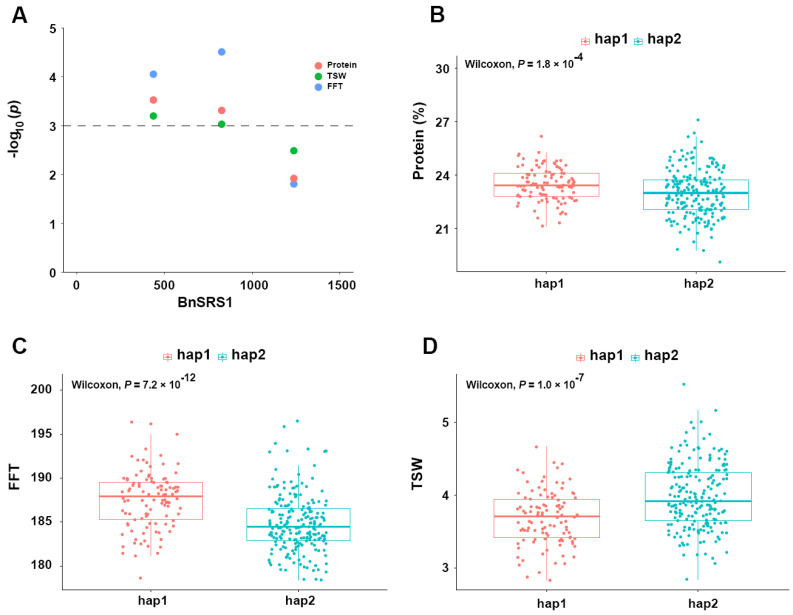
The association analysis between *BnSRR1* and agronomic traits in *B. napus*. (**A**) Manhattan plot with protein content, final flowering time (FFT), and thousand seeds weight (TSW). (**B**–**D**) Phenotypic analysis of different genotypic groups at significant loci for protein content, FFT, and TSW. There were 106 and 203 *B. napus* accessions in Hap1 and Hap2 populations, respectively.

Previous studies have demonstrated that *SRS* genes have an impact on various plant traits, including flower development [12], root development [2,4], plant height, etc [11,20,66]. To investigate the impact of *BnSRS* genes on agronomic traits in *B. napus*, a natural population of 324 *B. napus* accessions was employed, and the relationship between *BnSRS* and agronomic traits was analyzed [52]. After filtering, a total of 3,320,299 SNPs were identified from the SNP genotype data of these natural populations, out of which 168 SNPs were located on the 34 gene sequences, with 111 on the A subgenome and 57 on the C subgenome. Interestingly, the density of SNPs within the *BnSRS* genes was found to be higher in the A subgenome (5/kb) than in the C subgenome (2/kb), indicating an asymmetric evolution of the *BnSRS* genes between the A and C subgenomes (Table S7). However, the SNP density within the *BnSRS* gene (3.4/kb) was lower than the genome-wide SNP density (4.5/kb), implying its relative conservation. Furthermore, annotation of all the SNPs identified 86 SNPs in the exon region (including 48 nonsynonymous, 37 synonymous, and 1 splicing junction mutation). Remarkably, group II had the highest number of SNPs per *BnSRS* gene on average, approximately 15, followed by group III (12 SNPs per gene), and group I (11 SNPs per gene). The minimum in group IV showed only about 4 SNPs per gene. Additionally, significant differences were observed in the distribution of SNP numbers among duplicated gene pairs. For instance, *BnSRS31* had no SNP distribution, while 17 SNPs were identified in its corresponding duplicated gene, *BnSRS14*. Finally, an association analysis between SNPs and agronomic traits was conducted to examine the effect of the *BnSRS* gene on agronomic traits in *B. napus*. The results showed that a total of 27 SNPs were associated with at least one trait. For example, the gene *BnSRS1* was significantly associated with protein content, final flowering time (FFT), and thousand seeds weight (TSW) (Figure 10A). Furthermore, the *B. napus* population was divided into different subgroups according to the significant loci, the results showed that the phenotypic differences between subgroups were also highly significant (Figure 10B–D). These findings suggest that *BnSRS* genes play a significant role in regulating essential agronomic traits in *B. napus*. Additionally, the identified SNPs can serve as valuable genetic resources for future research exploring the functional properties of *BnSRS* genes.

## 4. Conclusions

In this study, we identified a total of 34 *BnSRS* genes and categorized them into four subgroups based on their phylogenetic relationships. Most members within each subgroup exhibit similar motifs, while variations in motifs among subgroups may contribute to the functional diversity of *BnSRS* genes. The presence of *cis*-acting elements in the *BnSRS* genes promoters, the expression patterns in different tissues and under various abiotic and biotic stresses, as well as the protein-protein interaction analysis indicated their potential role in regulating various aspects of plant growth, development, and adversity stress. Furthermore, association analysis revealed the involvement of *BnSRS* genes in the regulation of several important agronomic traits. Overall, These findings contribute significantly to our understanding of the *BnSRS* gene family and establish a solid foundation for further research on the biological and functional properties of *BnSRS* genes.

## Data Availability

The corresponding data are shown in Supplementary Materials.

## Supplementary Materials

The following supporting information can be downloaded at: https://www.mdpi.com/article/10.3390/genes14071421/s1, Figure S1: Multiple synteny among five species. A, B, C, D, and E represent rice, Arabidopsis, B. napus, cotton, and soybean, respectively; Figure S2: The abundance of the identified cis-acting elements in the promoters of BnSRS genes; Figure S3: The expression levels of BnSRS genes in silique (a) and seed (b) tissues. Table S1: Characteristics of physicochemical properties of the BnSRS genes.; Table S2: Ka/Ks analysis for duolicated gene pairs of BnSRS genes.; Table S3: The position of cis-acting regulatory elements of BnSRS genes.; Table S4: Predicted protein-protein interactions of SRS Proteins in B. napus.; Table S5: The GO enrichment analysis results of interacting proteins.; Table S6: The KEGG pathway analysis results of interacting proteins.; Table S7: SNPs of the SRS genes identified in 324 B. napus accessions.
