# Supplementary material for: Characterization and Potential Function Analysis of the SRS Gene Family in Brassica napus
Source: Genes (Basel). 2023 Jul 10;14(7):1421. doi: 10.3390/genes14071421 (PMC10379590; doi:10.3390/genes14071421)
Supplement: Supplementary file 1 [file genes-14-01421-s001.zip › genes-2479561-supplementary figures.pdf]

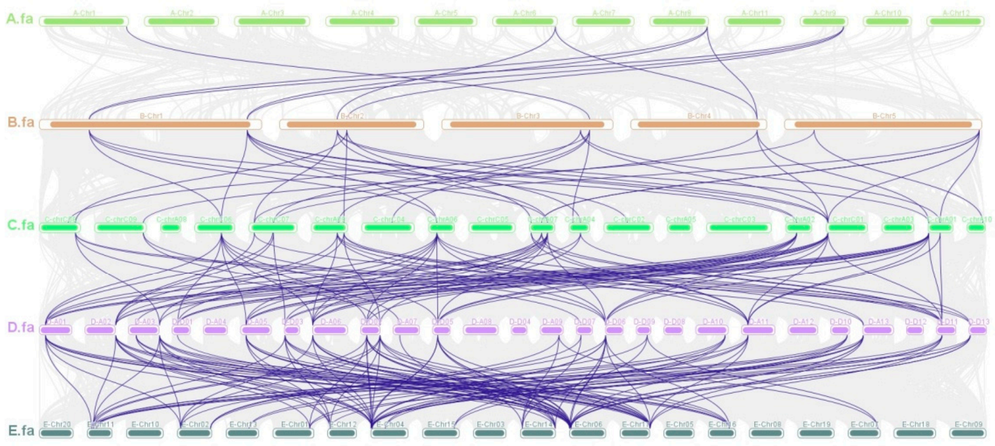

Supplementary Figure S1. Multiple synteny among five species. A, B, C, D, and E represent rice, *Arabidopsis*, *B. napus*, cotton, and soybean, respectively. The purple lines represent *SRS* genes that are homologous between two species.

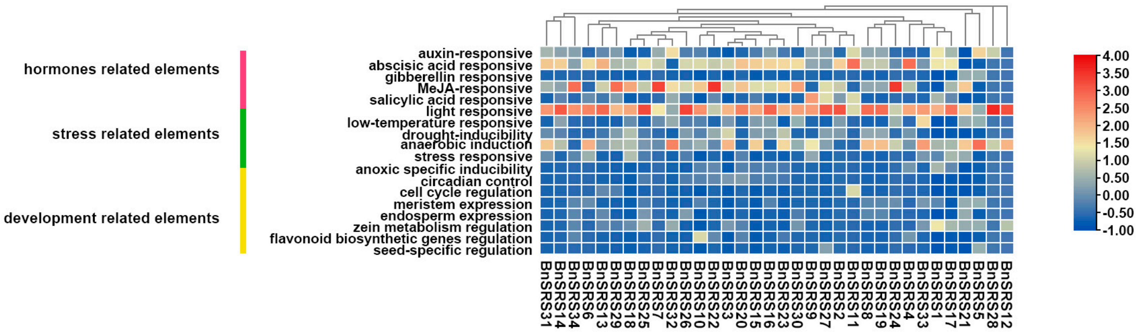

Supplementary Figure S2. The abundance of the identified *cis*-acting elements in the promoters of *BnSRS* genes.

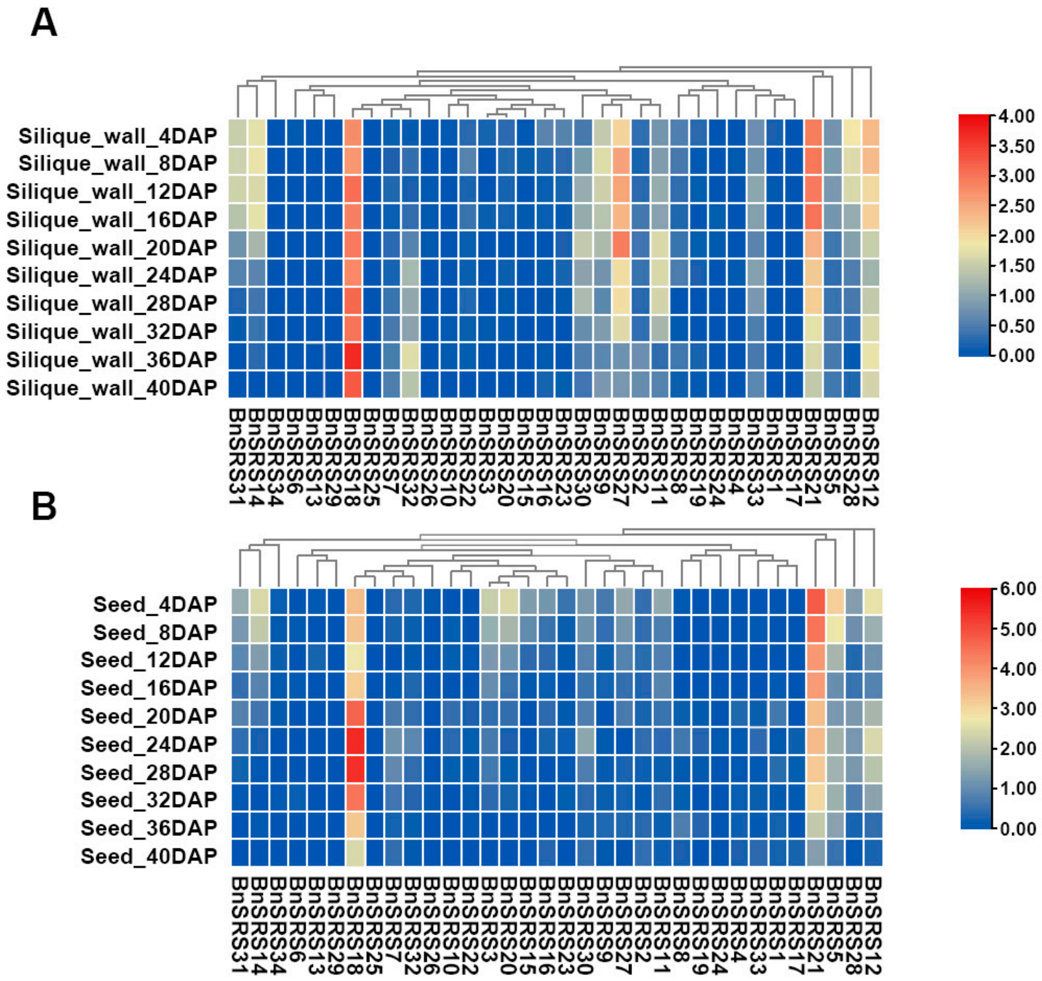

Supplementary Figure S3. The expression levels of *BnSRS* genes in silique (a) and seed (b) tissues.
